# Septic Arthritis of the Temporomandibular Joint Secondary to Acute Otitis Media in an Adult: A Rare Case with* Achromobacter xylosoxidans*

**DOI:** 10.1155/2017/3641642

**Published:** 2017-03-28

**Authors:** Ryan Chin Taw Cheong, Laura Harding

**Affiliations:** Broomfield Hospital, Mid Essex Hospital Services NHS Trust, Court Road, Broomfield, Chelmsford CM1 7ET, UK

## Abstract

Septic arthritis of the temporomandibular joint (SATMJ) is a rare complication of acute otitis media (AOM) with only four reported cases in the English and Japanese literature. Based on the unusual nature of this clinical condition, we discuss the first documented case due to* Achromobacter xylosoxidans* and the utility of myringotomy with long-term intravenous antibiotics via a peripherally inserted central catheter (PICC). We describe the case of a 76-year-old male patient that was brought in by ambulance to the accident and emergency (A&E) department due to severe right-sided otalgia with increased hearing impairment. A clinical diagnosis of acute otitis media with sepsis was made and the patient was commenced on the sepsis protocol. He then developed symptoms of septic arthritis of the TMJ which was confirmed on radiological imaging. After a multidisciplinary team discussion, the patient was treated with a myringotomy and intravenous ceftriaxone for 8 weeks in the community via a PICC rather than TMJ arthrocentesis with positive outcomes at 3 months' follow-up.

## 1. Introduction

Septic arthritis of the temporomandibular joint (SATMJ) is known to result in significant morbidity if diagnosis is delayed. It is considered to be a medical emergency and according to literature mortality rates can be up to 12% in the extremity joints, and up to 75% of survivors develop significant functional disability in the involved joints or growth problems [1]. The major cause of inoculation of the microorganism is usually a penetrating wound resulting from joint injection or direct extension from a contiguous site. Occasionally, the infectious organism gains access to the affected joint from a distant focus by haematogenous dissemination [2]. No clear consensus has been reached on the diagnosis and management of this condition. SATMJ is a rare complication of acute otitis media (AOM) with only four reported cases in the English and Japanese literature [1–4]. Of the reported cases, the offending organisms are the commonly found Group A streptococci and Methicillin-resistant* Staphylococcus aureus* (MRSA). Based on the unusual nature of this clinical condition, we discuss the first documented case due to* Achromobacter xylosoxidans* and the utility of myringotomy with long-term intravenous antibiotics via a peripherally inserted central catheter (PICC).

## 2. Case Presentation

We describe the case of a 76-year-old male patient that was brought in by ambulance to the accident and emergency (A&E) department due to severe right-sided otalgia with increased hearing impairment. The patient reported that his symptoms developed acutely within 2 days. He was pyrexic with tachycardia on presentation and right-sided cervical lymphadenopathy was palpable. The right pinna did not protrude from the mastoid and there were no signs of mastoid inflammation. On palpation over the mastoid process there was no fluctuance or pain. Otoscopy of the right ear demonstrated a clear external auditory canal, with an erythematous and bulging tympanic membrane. A clinical diagnosis of acute otitis media was made. There were no other significant findings on examination. The patient used a hearing aid on the right and had a dead ear on the left due to a congenital hearing defect with no other significant past medical history.

**Figure 1:**
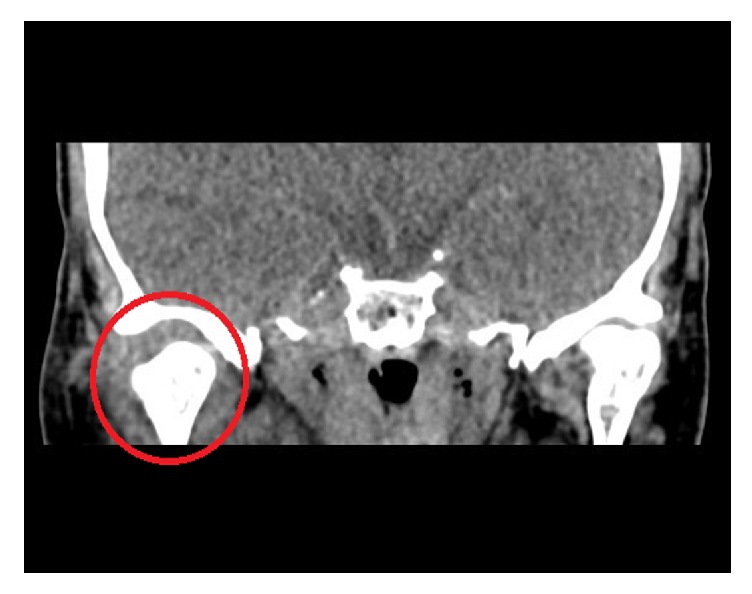
Coronal view.

Laboratory full blood count and biochemical profiling in the A&E department demonstrated an acute inflammatory process with a C-Reactive Protein value of 39.2 mg/L (normal 0−7.5 mg/L), White Blood Cell count value of 14.2 × 10^9^/L (normal 4–10 × 10^9^/L), and Neutrophil count of 12.6 × 10^9^/L (normal 2–7 × 10^9^/L). No aerobic or anaerobic microorganisms were isolated after 5 days of incubation in blood culture. The sepsis protocol was initiated and the patient was treated with 4.5 g of intravenous Tazocin (piperacillin and tazobactam) initially. Within 24 hours of admission under the otolaryngology team, the patient developed a right-sided fluctuant preauricular swelling, trismus with a maximal incisal opening (MIO) of 12 mm, and pain on neck movement. A clinical diagnosis of septic arthritis of the TMJ was made by the oral and maxillofacial consultant. Computed tomography (CT) scan of the temporal bones demonstrated soft tissue and infected effusion around the right TMJ (Figure 1) and mild anterior attic soft tissue thickening with soft tissue density in close proximity to the ossicles in the right middle ear. There were no significant radiological features in the mastoid.

**Figure 2:**
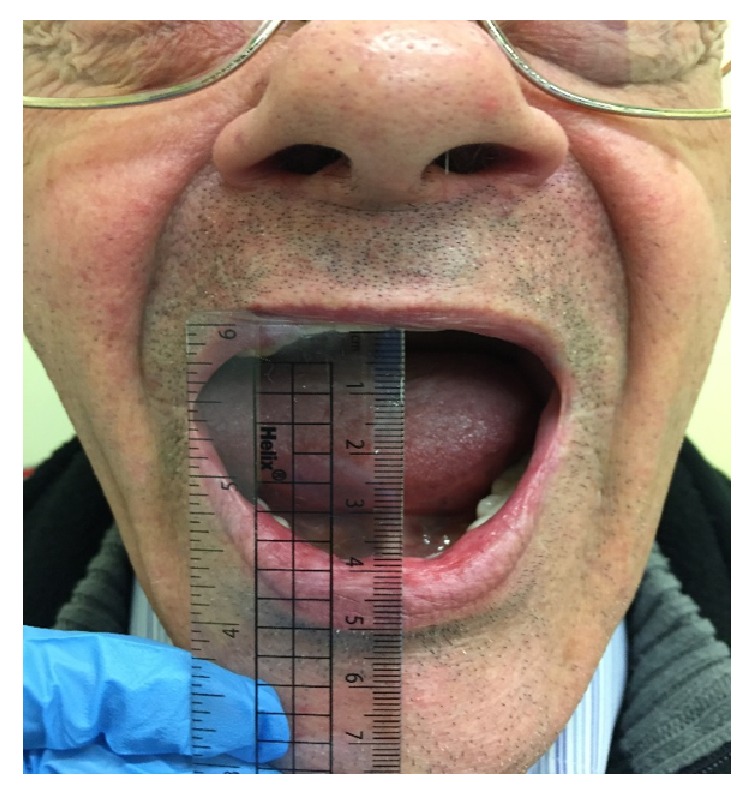
MIO of 38 mm at 3 months' follow-up.

A multidisciplinary team discussion between the consultant otolaryngologist, consultant oral and maxillofacial surgeon, and consultant microbiologist resulted in the following management plan. A myringotomy was performed to release the right middle ear fluid collection which cultured positive for* Achromobacter xylosoxidans* that was resistant to gentamicin. Arthrocentesis of the TMJ was not performed. Therefore, a diagnosis of septic arthritis is based on clinical suspicion and has not been demonstrated by aspiration and standard microbiological methods. The patient was discharge after 8 days of hospital admission and completed 8 weeks of IV ceftriaxone 2 g twice a day via a PICC in the community. Upon 3 months' follow-up, the patient made adequate functional recovery. The patient had improvement of trismus with an MIO of 38 mm (Figure 2) and no preauricular swelling was present. There was no residual functional disability or pain during mastication. Otoscopy revealed a normal right external auditory canal and normal tympanic membrane with a healed myringotomy scar and light reflex was present. Pure tone audiometry revealed that the patient's hearing had returned to his baseline function. The patient had declined aggressive range of motion exercises.

## 3. Discussion

A literature review was performed using electronic databases (PubMed, Medline) with the keywords “acute”, “otitis media”, “septic”, “arthritis”, “temporomandibular joint”, “Achromobacter xylosoxidans”, and manual cross-referencing between the literature. This yielded 4 manuscripts published reporting septic arthritis of the TMJ as a complication of otitis media in the English and Japanese literature. Bast et al. and Gayle et al. described paediatric cases [1, 3] and Cai et al. and Ishikawa et al. described adult cases [2, 4]. The offending organisms isolated in these cases were Group A streptococci and Methicillin-resistant* Staphylococcus aureus* (MRSA). The patients in all four cases were treated with TMJ arthrocentesis and IV antibiotics, with Bast et al. recommending additional mouth exercises.

Amos et al. discovered that the most common offending organism in septic arthritis of the TMJ in 33 patients was* Staphylococcus aureus* and* Streptococcus pyogenes* [5].* Achromobacter xylosoxidans* is an aerobic, motile, and gram-negative rod first described in 1971 by Yabuuchi and Ohyama, who discovered it in patients with chronic, purulent otitis media [6].* Achromobacter xylosoxidans* is a very uncommon cause of bacteremia that inhabits a variety of aqueous environments. In several large reviews of gram-negative bacteremia, no cases were due to* Achromobacter xylosoxidans* [7]. Bacteremic infection with this organism is thought to occur mostly nosocomially in immunocompromised patients and to be associated with a high mortality [8]. Treatment of* Achromobacter xylosoxidans* infections is often difficult and an optimal antimicrobial regimen has not been determined [8].

## 4. Conclusion

In the context of an* Achromobacter xylosoxidans* infection and to minimise the risk of deeper seeding of the infection we have opted to treat our patient with a myringotomy and prolonged course of IV ceftriaxone via a PICC rather than TMJ arthrocentesis with positive outcomes in an adult patient with septic arthritis of the temporomandibular joint as a complication of acute otitis media.
